# Hair Metal Levels and Childhood Weight Gain

**DOI:** 10.18502/ijph.v49i8.3895

**Published:** 2020-08

**Authors:** Mohsen VIGEH, Kazuhito YOKOYAMA, Takehisa MATSUKAWA, Atsuko SHINOHARA, Katsumi OHTANI, Mamak SHARIAT

**Affiliations:** 1. Maternal, Fetal, and Neonatal Research Center, Tehran University of Medical Sciences, Tehran, Iran; 2. Department of Epidemiology and Environmental Health, Faculty of Medicine, Juntendo University, Tokyo, Japan; 3. Occupational Epidemiology Research Group, National Institute of Occupational Safety and Health, Kawasaki, Japan

**Keywords:** Lead, Children, Gender, Weight, Hair

## Abstract

**Background:**

Exposure to toxic metals remains a public health problem with lifelong impacts on childhood growth and development. We aimed to investigate metals effects on preschool children’s anthropometric variables.

**Methods:**

The study was conducted in Tehran, Iran, from Jul 2013 to Mar 2016. We measured scalp hair metal concentrations (lead, cadmium, arsenic, zinc, manganese, and cobalt), using inductively coupled plasma mass spectrometry, in 207 preschool children’s (36 to 72 months old).

**Results:**

A significant negative correlation between children’s hair lead levels and children’s weight was found (r= −0.178, *P<0.05*). Linear regression analysis confirmed the relationship when adjusted for the confounders, including children’s age, sex, height, family income, and maternal education (β= −0.191; t= −3.426, *P*< 0.01). The ANOVA analysis showed a significant (*P*<0.01) difference between hair lead level and children’s weight-for-age percentiles. Totally and separately, in almost all weight percentiles, hair lead levels were higher in girls than boys.

**Conclusion:**

The present study on Iranian children showed the current levels of lead exposure might negatively influence on children growth, with higher risk for girls than boys.

## Introduction

After the Industrial Revolution, exposure to many of toxic substances, including metals, has relatively increased. Among toxic metals, lead is a well-known poison for humans’ organs. People of all ages encounter lead from the air, dust, soil, and drinking water, which it mostly absorbed through the gut (1). Furthermore toxic metals, deficiency and/or excess of essential elements may induce adverse health effects, such as children’s growth problems (2–4).

Prenatal and early childhood exposed to toxic metals remains a public health problem due to potentially impacts on children’s growth and development (5, 6). However, the effect size and ‘safe’ level have not been clearly known. For instance, after issued the new acceptable levels of blood lead for pregnant women and children (i.e., blood lead <5μg/dL) (7), researchers have claimed toxicity of lead the relatively lower blood concentrations (5, 8, 9).

Early childhood is a critical period of human growth and development, when many toxicants can induce the risk of several adverse effects. Studies on Iranian preschool children have found considerable percentage of underweight (7.5 to 20%) (10–13) and short stature children (6.6%) (10) compared with international data (14), with a significant difference between boys and girls (13). The reason(s) of Iranian preschool children growth problems has not been fully understood. Although socioeconomic status and subsequent diets insufficiency may play an important role in child’s growth insufficiency, other factors (i.e., genes, gender, and environmental pollutions) may involve as well. On the other word, “Iranian infants are exposed to relatively higher levels of metals from industrial activities, via polluted air and soils” (15, 16).

“Serial blood measurements may offer a better estimation of the body burden of metals than a single measurement, but they are usually impractical”. Instead, measuring metals in hair samples offers advantages for research and clinical screening / diagnosis tests, as an alternative to blood and/or urine sampling (17–19). It could present more information on cumulative level and non-invasive sample collection, especially when encounter occurs at medium to high level polluted areas (20). The current study, as a rare researcher design in Iran, measured concentrations of a set of toxic and essential metals together in pre-school children’s scalp hair to evaluate whether metals could associate with children’s anthropometric characteristics, at the current exposure levels. Thus, the researchers had a great opportunity to known about metals competition or synergism in absorption, accumulation, and toxicities in childhood.

## Materials and Methods

### Study participants

The present cross-sectional study was conducted in Tehran, Iran, from Jul 2013 to Mar 2016. Participant’s recruitment has been done when children brought for regular growth and development assessment and/or mandatory vaccination. We invited 265 children to the survey (via their parents), which 78% accepted our request. Two hundred and six preschool children (36 to 72 months old) who did not have chronic condition (cancer, gross birth anomaly, and so on) and lived in Tehran districts were recruited to the study. The Ethical Committees for the Vice-Chancellor of the Research Department and Institutional Review Board of Tehran University of Medical Sciences approved the study design and procedures. The study was conducted under the supervision of the Maternal, Fetal, and Neonatal Research Center, Tehran University of Medical Sciences, Iran. The study purpose and procedures were fully explained to the children’s parents, and the study was conducted with their informed consent. Participation in the study was strictly voluntary.

### Sample size calculation

The sample size was calculated by the following formula:

$$n=\frac{t^{2}\times p\left(1-p\right)}{m^{2}}=183$$

**n** = required sample size
**t =** confidence level at 95% (standard value of 1.96)
**p =** estimated prevalence of growth/mental problem in Child Development Review Manual (Harold Ireton, 2004) (5%)
**m =** margin of error at 10% (standard value of 0.1)

### Questionnaire and measurements

Participants’ characteristics (i.e., maternal age, education, and incomes) were gathered using face-to-face structured questionnaire that was developed for the current study. Children weights were obtained using a standard balance beam scale. With the children in standing position, height was measured to the nearest 0.1 cm using a rigid stadiometer. The children weight-for-age and height-for-age percentiles was obtained according to WHO guideline, separately for boys and girls (21).

### Collection and analysis of hair samples

For removing external contamination from children’s hair, mothers were asked to wash the children’s hair with shampoo or soap, then rinse with plenty of tap water (at least five minutes) prior to sampling. None of children used any type of hair treatment, at least in the past month. After cutting children’s hair (about 3 cm from the child’s scalp; 2–5 g), samples were placed in plastic packs and transferred to the laboratory for metals measurements.

For analysis concentration of metals (lead, cadmium, arsenic, zinc, manganese, and cobalt), hair samples were weighed (5–10 mg) and put into Teflon perfluoroalkoxy bottles, and 0.4 ml of concentrated nitric acid (ultrapure grade, Tama Chemicals Co., Kawasaki, Japan) was added. The bottles were left overnight at room temperature (18–28 °C). Then sample mixture digested with 0.2 ml of hydrogen peroxide (ultrapure grade, Tama Chemicals Co., Kawasaki, Japan) using a microwave oven (MLS-1200 MEGA, Milestone Srl, Bergamo, Italy). This process was done in five steps using various power levels set at 250, 0, 400, 650, and 250 W for 6, 1, 6, 6, and 6 minutes, respectively. Next, the volume of the digested sample was adjusted to 1.0 ml using ultrapure water. After dilution with 0.5% nitric acid solution, subsequent measurements for metal concentrations were done by inductively coupled plasma mass spectrometry (ELAN 6000, PerkinElmer, Waltham, MA, USA) using multi-element standard solutions XSTC-13B and XSTC-622 (SPEX CertiPrep, Metuchen, NJ, USA). Each measurement was repeated three times, and the average of the three measurements was used for statistical analyses. For instrument calibration throughout the measurements, at least 10% of the analyses were external standards, and 5% were blank (pure water).

### Data analysis

To reduce the influence of outliers and normalize residual distribution, we used the common logarithm (log_10 _) of blood lead in the statistical analysis. Pearson correlation coefficient was used to study relationships between hair metal levels with children’s weight, height, and age. The ANOVA test was employed to study difference of metal levels and children’s weight percentiles (<3, 3–15, 16–50, 51–85, 86–97, and >97). The Student’s *t*- test compared metals levels and anthropometric characteristics between boys and girls. The same statistical analysis was employed to camper low percentiles (<50, n=107) with high percentile (>50, n=100) of weight-for-age. Linear regression analysis was used to examine the relationship between hair lead levels and child’s weight, controlling for possible confounding variables, such as children’s age, sex, height, family income, and maternal education. Statistical analyses were performed using SPSS (IBM SPSS, Armonk, NY, USA), and statistical significance was determined using (*P*<0.05).

## Results

**Fig. 1:**
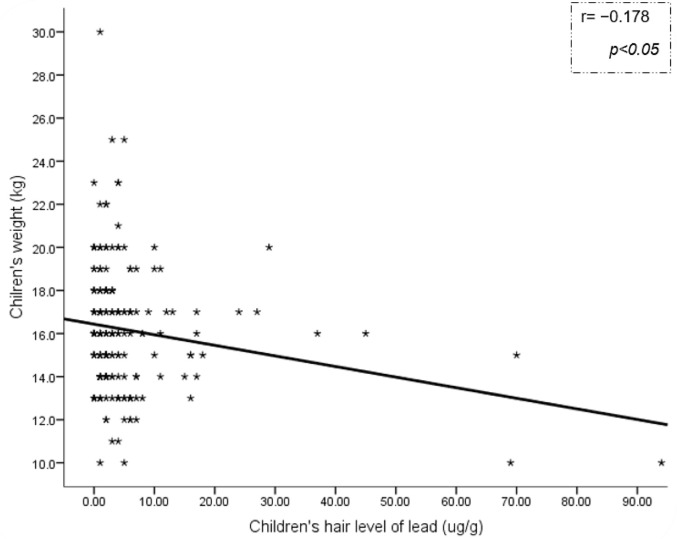
Pearson correlation between hair levels of lead and children weight

Basic statistical analysis of continuous and categorical variables and children’s hair metal levels was shown in the Table 1. There was 207 participants’ data for statistical analysis (89 boys and 118 girls). Due to technical problems (undetectable metal levels and/or no matching with standards/blank samples) some of metal measurement’s data were not included in statistical analyses (3 for zinc and cobalt; 4 for lead, cadmium, and manganese; and 31 for arsenic). Among all potentially toxic metals, mean lead concentration was relatively higher (mean: 5.75 μg/g) (Table 1). Pearson’s correlation coefficient showed a significant negative correlation between children’s hair lead levels and weight (r= −0.178 and −0.149, for original and log_10_ transformed, *P*< 0.05) (Fig. 1). Linear regression analysis confirmed a significant negative relationship between children’s hair lead and weight when adjusted for the confounders (β= −0.191; t= −3.426, *P*<0.01) (Table 2). Pearson’s correlation coefficient revealed significant positive correlations between levels of lead with cadmium (r= 0.565, *P*<0.01) and lead with arsenic (r= 0.565, *P*<0.01).

**Table 1:** Mean, standard deviation (SD), and range of continues variables and number of “yes” answer with percentage of categorical variables of the study participants (n=207)

***Continuous variables***	***Mean ± SD***	***Median***	***Range***
Children’s			
Age (month)	52.1 ± 10.9	50.0	36 – 72
Weight (kg)	16.72 ± 2.93	16.5	10.4 – 30.0
Height (cm)	104.8 ± 7.7	105.0	86 – 126
Body mass index	15.3 ± 2.1	15.2	10.5 – 23.0
Maternal weight (kg)	32.6 ± 5.0	32	21 – 46
Paternal weight (kg)	37.7 ± 5.8	37	26 – 56
Level of metals in child hair (μg/g)			
Lead	5.75 ± 10.80	2.77	0.34 – 94.87
Cadmium	0.11 ± 0.17	0.60	0.004 – 1.69
Arsenic	0.06 ± 0.09	0.04	0.00 – 0.91
Magnesium	0.91 ± 2.18	0.52	0.08 – 20.79
Zinc	132 ± 124	121	6.1 – 1593.5
Cobalt	0.07 ± 0.02	0.02	003 – 2.57
*Categorical variables*	*No*		*%*
Childs’ sex (boy)	89		43
Education ^*^			
Senior high school	46		22
Junior high school	111		54
college & university	50		24

* Seven missed data

**Table 2:** Examine the relationship between hair lead levels and child’s weight using the linear regression model; controlled for possible confounding variables^*^ (n=201)^†^

***Variables***	***Coefficients***		***t***	***P value***
	***Unstandardized (B) Standardized (β) (95% CI)***			
Hair lead level (g/ug)	−0.051 (−0.080 – −0.022)	−0.191	−3.426	0.001
Children’s age (month)	0.063 (0.021– 0.105)	0.236	2.944	0.004
Children’s height (cm)	14.80 (8.659 – 20.957)	0.391	4.750	< 0.001
Children’s sex (girl)	−0.892 (−1.556 – −0.227)	−0.153	−2.645	0.009
Maternal education	0.151 (−0.123 – 0.424)	0.139	1.088	NS
Family income (IRR)	0.001 (−0.001– 0.002)	0.066	1.207	NS

* Children’s age, sex, height, family income, and maternal education

† There was 6 missed data for the analysis

NS: none significant

**Fig. 2:**
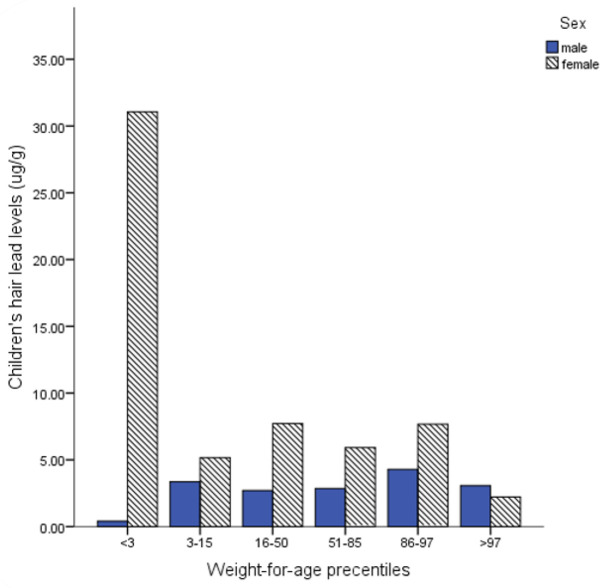
Differences between girls and boys of hair lead levels among children’s weight-for-age percentiles

The ANOVA analysis showed significant difference (*P*<0.01) in mean concentration of hair lead and children’s weight-for-age percentiles (23.4μg/g for percentile <3, 4.6μg/g for percentile 3–15, 6.0μg/g for percentile 16–50, 4.3μg/g for percentile 51–85, 5.7μg/g for percentile 86–97, and 2.8μg/g for percentile >97), with highest concentration difference (20.6μg/g) between percentiles <3 and >97. In all percentiles, except for >97, mean levels of hair lead were higher in girls than in boys (Fig. 2).

The Student’s t-test showed a significantly higher mean hair lead levels in children at lower weight- for-age percentile (<50, n=107) compared with higher percentile ones (>50, n=96) (mean: 6.9μg/g and 4.4μg/g, respectively). Except for cobalt, the mean of all metals was higher in girls than boys (only significant for lead <0.01), and anthropometric characteristics (weight and height) were lower in girls than boys (Table 3).

**Table 3:** Comparison of hair metal levels and anthropometric characteristics between boys and girls (mean ± SD)

***Variable***	***Boys N=89***	***Girls N=118***	***P value ^a^***
Age (month)	52.9 ± 10.7	51.6 ± 11.0	NS
Weight (kg)	17.8 ± 3.0	15.9 ± 2.6	< 0.01
Height (cm)	106.6 ± 7.6	103.4 ± 7.6	< 0.01
Body mass index (kg/m^2^)	15.6 ± 2.0	15.1 ± 2.1	0.052
Level of metals in child hair (μg/g)			
Lead	2.95 ± 2.64	7.77 ± 13.65	< 0.01
log_10_	0.31 ± 0.39	0.60 ± 0.45	< 0.001
Cadmium	0.08 ± 0.12	0.12 ± 0.19	NS
Arsenic	0.06 ± 0.07	0.07 ± 0.10	NS
Magnesium	0.68 ± 1.55	1.07 ± 2.54	NS
Zinc	126.2 ± 60.0	137.7 ± 156.1	NS
Cobalt	0.09 ± 0.29	0.06 ± 0.09	NS

a Student t-test

NS: none significant

Among other metals manganese was significantly correlated with children’s height (r=0.202, *P*<0.01). However, this relationship was not significant when we adjusted height-for-age and in the multivariate analysis.

## Discussion

We found a negative correlation between hair lead levels and weight in preschool children. This relationship was confirmed by linear regression analysis when adjusted for possible confounders. When the study adjusted weight for age and sex, the mean of hair lead level was lower in children at higher percentiles of weight than in the lower percentiles. Overall, weight and height were higher and metals levels, exception for cobalt, were lower in boys than in girls.

Impact of hair lead concentration on children growth has been reported by an Italian study (20). A study on 246 children (3–8 yr) reported negative correlations between blood and bone lead levels (7.30 μg/dL and 0.69 μg/g, respectively) with children’s height and weight (22). Similarly, the NHANES data (1999–2006) has found an inverse association between children weight and blood lead levels (OR=0.67) (23). Additionally, 15 gram decline in children’s weight per one unit increased in maternal blood lead (mean 5.6 μg/dL) (24), or 131 gram weight decreasing by increasing one standard division in maternal patella lead (μg/g) have been reported (25). On the other hand, several studies have showed significant relationships between prenatal blood lead and birth weight reduction (5, 26, 27). In addition, negative associations between the prenatal blood lead exposure with childhoods’ weight-for-age, weight-for-length, head circumference, and upper arm circumference have been observed (28, 29). Similarly, comparison between two studies on children (ages 2–12) over two decades in Dallas, Texas, revealed a 3.5 kg greater children weight by decreasing in mean of blood lead levels from 23 to 1.6 μg/dL (30). Thus, lead in different biological media and exposure period may adversely effect on children growth.

Among other measured metals in the current research only manganese significantly correlated with children’s weight. However, this relationship did not remain significant after controlling for children age. Several studies have reported adverse effects of manganese on intrauterine growth and birth size, usually in a nonlinear fashion (inverted U-shaped curve relationship) after adjusting for potential confounders (3, 4, 31,32). Thus, this element effects on child growth need to be studied more in the future researches.

The current study results showed higher levels of some metals in girls’ than boys’ hair. Previous studies have reported accumulation of metals related to participants’ gender (33–36). For example, studies in Iran and Italy have reported higher level of scalp hair elements (i.e., lead, cobalt, cadmium, copper, and zinc) in girls than in boys (36, 37). In addition, some researchers have revealed higher blood levels of manganese, copper, arsenic, cadmium and selenium in females than males (38–40). Similar to hair and blood, higher levels of urinary cadmium in females than in males was shown (33). On the other hand, some studies on children have reported lower blood levels of lead in girls than in boys (32, 34). These differences in metals levels between girls and boys could be relate to female vulnerability to absorb metals and/or a sex-related metabolic differences of metals, such as a higher bone release of lead in exchange with calcium (41) or shorter growth period, due to complete skeletal growth generally earlier and faster, in girls than in boys (37, 38). Thus, children’s gender may play an important role in metal accumulation and consequently on their toxicities (42, 43).

Environmental pollution could be the main source of exposure in Tehran, as the city has considerable numbers of different industries and large cars traffics. Although concentrations of many elements are lower in residential zones than in industrial areas, some elements are present in the same (arsenic and zinc) or even higher levels (manganese, antimony, and copper) in these zones of Tehran (15). In addition, some heavy metal contents were reported to be higher along the city roads (i.e., cadmium, 4 mg/kg; lead, 669 mg/kg; zinc, 614 mg/kg) (16) than the acceptable levels in natural soils (cadmium, 3 mg/kg; lead, 100 mg/kg; zinc, 300 mg/kg) (44).

There were some limitations for the current study. First, the participant recruitment and sampling were done in different children’s ages (from 36 to 72 months), which can potentially influence on the results. For reducing this factor effects, we adjusted weigh-for-age, separately for boys and girls. Second, although the participants washed their hair before sampling, the hair samples washing did not include the measurement procedure. Third, we had limited information on the children’s diets and insufficiently reliable data of fathers’ anthropometric characteristics. Finally, because the present research was a cross-sectional study, we cannot conclude causality effects of lead on children weight.

## Conclusion

The present study on Iranian children found a negative relationship between hair lead levels and child weight gain. Thus, lead, at the current exposure levels, might influence children growth. In addition, girls showed higher levels of hair metals than boys. Thus, gender may play an important role in the absorption, accumulation, and/or toxicity of metals. The future studies should consider children gender as a confounder whenever investigates metals toxicity. In addition, the findings can introduce a non-invasive method of sampling, instead of blood sampling, for measurement of metals exposure in children.

## Ethical considerations

Ethical issues (Including plagiarism, informed consent, misconduct, data fabrication and/or falsification, double publication and/or submission, redundancy, etc.) have been completely observed by the authors.
